# A modified diet to support conservation of the Atala hairstreak butterfly (*Eumaeus atala* Poey)

**DOI:** 10.1002/zoo.21628

**Published:** 2021-06-14

**Authors:** Elizabeth Braatz, Jamie Sincage, Zachariah J. Gezon, Lily T. Maynard, Amanda Ardente, Anne Savage, Kathleen E. Sullivan, Shannon Livingston, Eduardo V. Valdes

**Affiliations:** ^1^ Disney's Animals, Science, and Environment The Walt Disney Company Lake Buena Vista Florida USA; ^2^ The Butterfly Biosphere Thanksgiving Point Institute Lehi Utah USA; ^3^ Global Conservation Cincinnati Zoo & Botanical Garden Cincinnati Ohio USA; ^4^ Ardente Veterinary Nutrition, LLC Alachua Florida USA; ^5^ Proyecto Titi, Inc. Orlando Florida USA

**Keywords:** Atala hairstreak butterfly, butterfly, caterpillar, conservation, coontie, cycad, *Eumaeus atala*, *ex situ*, Florida, host plant, husbandry, modified diet

## Abstract

Raising insects in a laboratory for release into the wild is a common conservation practice, but maintaining breeding colonies year‐round can be limited by seasonal food availability. Food availability is particularly challenging for insects which depend on specific host plants. For example, our early efforts to rear the imperiled Atala hairstreak butterfly (*Eumaeus atala* Poey) resulted in colony failure during winter due to lack of food. To overcome this barrier, we developed a modified freeze‐dried host plant diet to support the colony. The diet consisted of reconstituted freeze‐dried leaves and stems from fresh‐growth coontie (*Zamia integrifolia*), the host plant for the Atala butterflies. We fed larvae less than 9 mm on this freeze‐dried diet and transferred them to live coontie plants after they were more than 9 mm. We reared a colony of Atala butterflies using these methods for 859 days, resulting in more than 3400 animals released into the wild. Comparing colony counts during that time period to the 548 days we reared them without modified freeze‐dried diet showed a clear benefit in using freeze‐dried diet. A growth trial (*N* = 40) of larvae fed on only freeze‐dried diet compared to larvae fed on fresh coontie cuttings found no significant difference in larval or pupal development between groups (*p*  =  0.71 and *p*  =  0.47, respectively). We, therefore, conclude that the freeze‐dried diet provided an appropriate alternative for Atala colonies when fresh growth from the host plant is unavailable, and we recommend use of this technique for raising other host plant‐dependent insect species of conservation concern.

## RESEARCH HIGHLIGHTS

- When rearing butterflies ex situ for conservation, many species of butterflies are highly host‐plant specific, and it can be difficult to provide enough food for them during nongrowing seasons
- After a colony failure due to food shortage, we created a modified freeze‐dried diet for the imperiled Atala hairstreak butterfly (*Eumaus atala*) using methods that could potentially be replicated for other butterflies
- The diet was highly successful, leading to the rearing and release of over 3800 adult butterflies back into the wild
- Key to the methods was using pure, new‐growth host plant, and switching larger larvae back to mature leaves

## Graphical abstract

Graphical abstract created by Jennifer Torchalski, Disney's Animals, Science and Environment, Lake Buena Vista, Florida, USA.

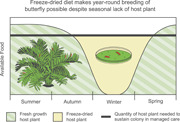

## 1 INTRODUCTION

Insect populations around the world are in steep decline, resulting in reduced biomass and in some cases extinction. For example, a 2017 study found that flying insect biomass in protected areas throughout Germany had declined 76% in just 27 years (Hallmann et al., 2017). Such declines are happening globally. A recent review of long‐term insect surveys from the past 40 years revealed that 40% of insect species worldwide are threatened with extinction (Sánchez‐Bayo & Wyckhuys, 2019). Of these, the order Lepidoptera (butterflies and moths) was identified as one of the most vulnerable taxa affected (Sánchez‐Bayo & Wyckhuys, 2019).

Conservation of imperiled butterfly species will likely require a multifaceted approach, including habitat restoration and management, population monitoring and management, and organism translocation and reintroduction (Crone et al., 2007; Daniels et al., 2018; Schultz et al., 2008). A review of 50 vulnerable butterfly species across Europe and North America found that the majority of conservation strategies recommend species reintroductions (Schultz et al., 2008, pg. 1), and that captive rearing should be used to maintain severely at‐risk populations, at least in the short term (Crone et al., 2007, p. 103). Many institutions have reared rare butterflies ex situ, including the National Park Service, University of Florida, Minnesota Zoo, San Diego Zoo, Toledo Zoo, and many more (e.g., Grimes, Harbor, and Us; Miami Blue Butterflies, 2017; Quino Checkerspot Butterfly Recovery & San Diego Zoo, 2017; Saving Prairie Butterflies and Minnesota Zoo, 2015).

One key and often difficult part of rearing butterfly colonies is providing the appropriate food to each species, which can be species and life‐stage specific. Many Lepidoptera larvae are specialists, capable only of eating very specific taxa or “host plants.” Of the 34 species of threatened or endangered butterflies in the USA, only two are not highly specialized eaters (USFWS Environmental Conservation Online System, 2020). Loss of host plants can result in butterfly extinction. For example, the Xerces blue butterfly was driven to extinction due to loss of habitat among the sandy sites that provided its host plant (Florida Museum, 2018). Maintaining reliable supplies of rare or seasonally dependent host plants is a challenge in the managed care of butterfly colonies.

The present study explored the effect of using processed and stored host plant material on the rearing and development of Atala hairstreak caterpillars in a laboratory setting and we present possible methods to overcome a seasonal dearth of the host plant supply to support a butterfly colony year‐round. In particular, we focused on the creation of a modified freeze‐dried diet to support the ex situ propagation and release of Atala hairstreak butterflies (*Eumaeus atala* Poey).

### 1.1 Atala case study

The Atala hairstreak butterfly (*E. atala*) in the continental USA is restricted to a single species of host plant for oviposition and larval consumption: *Zamia integrifolia* (synonyms include *Zamia pumila* and *Zamia floridana*), commonly known (and hereafter referred to) as “coontie.” Atala butterflies are able to use some other species of cycad, such as *Zamia erosa* (M. Feather, personal communication), but coontie is the only native cycad for Atala butterflies in the continental United States. The starchy root of coontie is edible, and around the turn of the twentieth century, European settlers greatly overharvested coontie for food (Koi & Daniels, 2015). When overharvesting led to the disappearance of Florida coontie from the landscape in the 1940s, the butterflies disappeared with them, and by the 1960s the butterflies were thought to be extinct (Koi & Daniels, 2015). Fortunately, a single remaining colony of butterflies was found along the coast of Miami, Florida, USA in 1979 (Koi & Daniels, 2015).

Many organizations and individuals have worked to conserve the Atala butterfly, both by increasing coontie availability in the wild and by developing techniques to rear Atala in managed settings (Koi, 2008; Koi & Daniels, 2015; Emmel & Minno, 1993; Ramírez‐Restrepo et al., 2017). Atala populations have risen over the past 20 years thanks to these conservation efforts, including a 2000 ban on commercially harvesting the plant for food, and the insect is no longer on the brink of extinction (Koi & Daniels, 2015). In collaboration with the University of Florida, Zoo Miami, and commercial farmer Dan Dunwoody, Disney's Animals, Science and Environment joined in the effort to repatriate Atala butterflies by breeding Atala butterflies in Orlando and shipping adult insects to partners for release into the wild.

Contributing to the Atala butterfly conservation effort requires maintenance of a year‐round breeding colony, which is limited by host plant availability (Koi & Daniels, 2015). The butterflies appear multivoltine in the southern tip of Florida and seasonal in their more northern ranges (Koi & Daniels, 2015). In south Florida, coontie can produce fresh growth year‐round (Koi & Daniels, 2015). Further north in the state, coontie does not produce fresh growth during the colder months (personal observation). Newly hatched larvae are not physically able to consume mature coontie leaves, which are thick, tough, and leathery. Thus, access to soft, fresh growth is critical to colony survival. We attempted to use greenhouses to stimulate fresh coontie growth but were unable to produce enough fresh growth to support an Atala colony during the winter. Even if the greenhouse method had worked, using greenhouses can be cost prohibitive. Conversely, outdoor plants produced large amounts of fresh growth during their natural spring/summer growing cycle. If it were possible to preserve the summertime fresh growth through the winter, it would be extremely useful for our work with the conservation of the Atala butterfly.

To increase our conservation impact of the Atala butterfly, we aimed to create a modified, easily storable diet that could support a year‐round colony of Atala larvae. Artificial or modified diets have been developed for other many other insects in managed care to avoid food shortages (e.g., Ahmad et al., 1989; Wu & Gong, 1997; Greene et al., 1976; Perkins, 1973). The methods used in this paper are an iteration upon a previous attempt which used commercially available Lepidoptera diet and was met with extremely limited success (Ardente et al., 2017). As an alternative approach, we attempted to use a freeze‐dried fresh‐growth coontie leaves to create the modified diet. This paper details the new methodology and provides an assessment of its success. We believe that the use of freeze‐dried host plant could be widely applicable to other species beyond Atala larvae, and it could have broad conservation impacts for specialist herbivorous insects in managed care.

## 2 MATERIALS AND METHODS

### 2.1 Study animals

This study was conducted using a colony of Atala butterflies in managed care in the invertebrate husbandry building at Disney's Animal Kingdom over the course of nearly 4 years. The colony was a donation from the University of Florida but was originally collected from the wild, and wild‐caught butterflies are occasionally added to the colony to maintain genetic diversity. The colony size averaged 273 individuals, with a median of 235 individuals. The colony reached a maximum size of 974 individuals, and a minimum size of 0. It should be noted that throughout this time period, adults were occasionally shipped out of the colony to Zoo Miami and Disney's Vero Beach Resort for release into the wild to reinforce the wild population of butterflies. Colony counts were based on a census of all individuals performed every other day throughout the study period.

### 2.2 Husbandry

#### 2.2.1 The colony

The protocol and procedures employed herein were ethically reviewed and approved by Disney's Animal Care & Welfare Committee (Disney's internal) using the Research Proposal Form designed by the AZA Research and Technology Committee. These methods describe our current rearing practices for Atala butterflies, which we applied through the duration of this study (from October 2017 to March 2020). Before the development of the freeze‐dried diet described in the present manuscript, we fed the colony live coontie plants only. Since the development of the freeze‐dried diet, we use fresh growth coontie whenever it is available, and freeze‐dried diet as needed when fresh growth is not available (i.e., winter).

We raised the insects in a temperature and humidity‐controlled environment (temperature: 25.5°C–28.9°C, humidity: 40%) in the invertebrate husbandry building at Disney's Animal Kingdom (Lake Buena Vista, Florida, USA). The invertebrate husbandry building uses the following light schedule: white light from 0700 to 1700, red light from 1700 to 1930, and dark from 1930 to 0700. We collected fresh growth coontie during the optimal portion of the growing season (May–December in Lake Buena Vista, FL). Fresh growth is new plant material that has grown within, approximately, the prior 2–3 weeks. It can be distinguished by its light shade of green and relative softness compared to other parts of the plant. In total, we harvested enough fresh growth coontie over one summer to create 1221 g of freeze‐dried food for winter use. We cut the fronds off at the base plant near the soil using gardening sheers, placed the fronds in plastic sealable freezer bags, and placed the bags in a walk‐in freezer at −17.8°C to −6.7°C for at least 24 h. The frozen plants were lyophilized (freeze‐dried) using a Virtis Genesis SQ 35XL freeze dryer system (SP Scientific). The plants were brought from −50°C to room temperature over a 60‐h period at a consistent vacuum pressure of approximately 300mTorr. Freeze‐dried plants were crushed into a fine green powder (resembling all‐purpose flour, with some remaining plant parts) with a Ninja Mater Prep Professional QB1004 food grinder (SharkNinja Operating LLC.). To maximize longevity, we stored the freeze‐dried powder in sealed plastic bags at −28.9°C. Photos of the final powder can be found in Appendices and Supporting Information.

Before feeding the modified freeze‐dried diet to Atala larvae, we rehydrated the pulverized, freeze‐dried coontie powder with water using a ratio of 1 g dried coontie powder to 4.25 g water, resulting in a thick paste. Although leaf moisture has been shown to be a critical component affecting the growth rate of insect larvae (e.g., Finke and Scriber, 1988; Scriber, 1979; Scriber & Feeny, 1979), the ideal ratio of pulverized diet to water was determined through trial and error, striking the balance of a wet enough paste to stay hydrated, but not too wet as to drown first instar larvae. We did, however, aim to stay close to the natural moisture content of fresh growth coontie leaves (80.9%, Appendix 1). To avoid mold, small batches of modified diet were made every 3–4 days. We stored the freeze‐dried diet in the refrigerator at 9°C for a maximum of 4 days. Photos of the setup and materials can be found in the Appendices and Supporting Information.

We offered the freeze‐dried diet to small (0–7 mm) atala larvae. We spread 0.5–1.0 tsp. of the paste at a thickness of 0.5 cm onto upturned 8 oz. natural polypropylene jars or “feeding trays” (Qorpak, Bridgeville, PA 15017) (see photos in Appendices and Supporting Information). Each day we used “bridges” of either mature coontie leaves or wooden sticks to encourage the larvae to crawl on their own from the old feeding tray to the fresh one (see photos in Appendices and Supporting Information). Larvae walked over the bridges to the fresh feeding trays, and the used trays were removed. We used soft, damp brushes to transfer any larvae that remained or wandered in the wrong direction.

After the Atala larvae became larger than approximately 9 mm, we moved them to a diet of mature coontie leaves. By restricting the use of the modified freeze‐dried diet to the smallest larvae, we increased efficiency and reduced labor costs for diet preparation. After the insects completed metamorphosis, we placed the adult butterflies in a flight cage with a coontie plant to encourage egg laying and an artificial nectar feeder (see photos in Appendices and Supporting Information). Note that we eventually replaced the whole plant with sprigs. We try to keep at least 60 adult Atala butterflies in the adult flight enclosure, and the enclosure also has lights and a fan to promote air circulation (Ardente et al., 2017; Harrison et al., 2019). We censused colony larvae, pupae, and adults once each week. We counted larvae after they were larger than 9 mm because smaller larvae are difficult and time consuming to see and count. We checked egg hatch rates by counting the number of eggs laid versus eggs hatched across 1108 eggs.

#### 2.2.2 Experimental cohort

Before changing the entire colony to a new diet, we reared an experimental growth trial group of 40 small (<5 mm) larvae. Half (*N* = 20) were randomly assigned to an experimental group and half (*N* = 20) were assigned to a control group. Larvae in the experimental group were fed only hydrated freeze‐dried diet. We reared them using the same methods described previously: we spread food paste onto upturned polypropylene jars and used “bridges” to encourage the larvae to crawl from the old feeding tray to the fresh one. The 20 larvae in this preliminary experimental group were never transferred to a diet of coontie clippings and were instead fed the freeze dried diet until pupation. Larvae in the control group were reared using the exact same methods and conditions as the experimental group but were only fed clipped sprigs of fresh‐growth coontie (see photos in Appendices and Supporting Information). The sprigs of coontie were kept fresh and hydrated using floral water picks. We counted larvae, pupae, and adults each day during the experimental trial.

### 2.3 Analyses

We calculated summary statistics using the colony count data. For the growth trial of 40 larvae, we used R version 3.4.0 statistical software (R Core Team, 2017) to test for differences among the number of animals in each treatment (experimental vs. control) that passed from larvae to pupae, pupae to adult, and total survival (i.e., three separate analyses) using Fisher's exact test. We used Fisher's exact test instead of a *χ* ^2^ test due to sample size. To test whether the overall pattern of insect pupation, emergence, and survivorship was significantly different between the control and experimental groups, we compared the number of insects that successfully pupated, emerged, or survived in each group simultaneously. Since this sample was slightly larger, we applied Pearson's *χ* ^2^ test of independence.

## 3 RESULTS

### 3.1 Colony population

**Figure 1:**
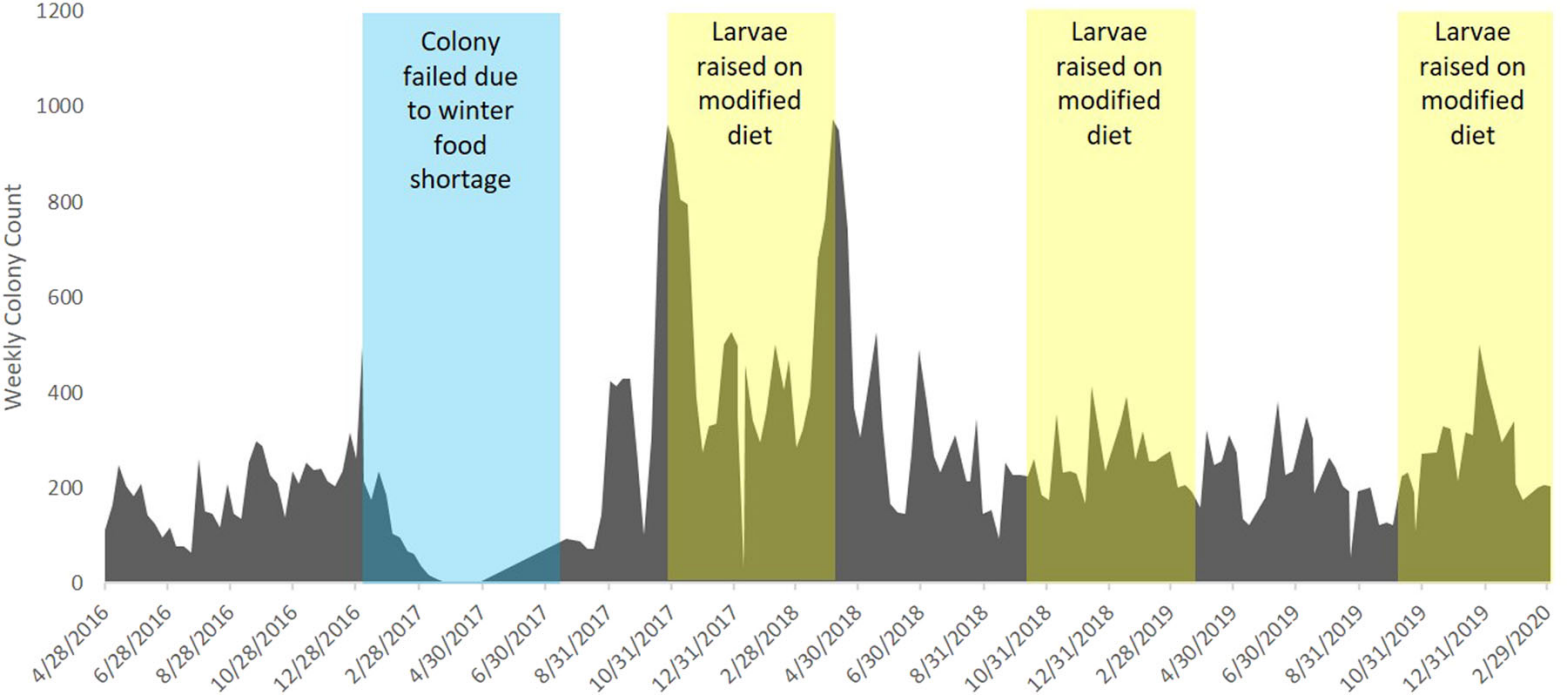
Weekly colony counts over time [Color figure can be viewed at wileyonlinelibrary.com]

By supplementing the colony with freeze‐dried diet, we were able to rear and release 3847 adult butterflies into the wild. Without the diet, we released 974 adults into the wild before the colony failed in 2017 (Figure 1). After 2018, we actively managed the population to keep the overall total under 1000 individuals. This slight reduction in numbers was an active management choice and was not due to the modified freeze‐dried diet or the animals' conditions. Approximate egg hatching rate was 51%, which is typical of Atala butterflies (personal observation).

### 3.2 Growth trials

A total of 75% of the larvae in the control treatment (raised on fresh coontie sprigs) pupated, spending a range of 14–16 days in the larval stage (Table 1). Similarly, the larvae fed the freeze‐dried diet spent a range of 15–22 days in the larval stage and 80% successfully pupated, although the difference was not statistically significant (Table 1). The percentage emergence rates for the control and experimental groups, respectively, was 87.5% and 100%. Total survivorship from larva to adult was similar between the control and experimental groups at 75% and 70%, respectively. Both groups had a high pupation and emergence success rate. There was no difference in overall pupation, emergence, or survivorship between the control group and the freeze‐dried diet group (Table 1).

**Table 1:** Atala butterfly larvae raised on the freeze‐dried diet developed slightly more slowly than insects fed fresh host plant cuttings, but there was no difference in survival

Description	Fresh plant diet	Freeze‐dried diet	*χ* ^2^ Results
Successful pupation	75%	80%	*χ* ^2^_1_ = 0.14
			*p* = 0.71
Successful eclosure	100%	88%	*χ* ^2^_1_ = 0.47
			*p* = 0.49
Total success (larvae to adult)	75%	70%	*χ* ^2^_2_ = 0.14
			*p* = 0.93
Days spent as larva	14–16	15–22	N/A
Days spent as chrysalis	13–14	14–17	N/A

## 4 DISCUSSION

Ex situ rearing of the Atala hairstreak butterfly and other Lepidopteran specialists is highly dependent on having a reliable food source, and our colony numbers suggest that our methods were successful in sustaining a colony of Atala butterflies. The purpose of this study was to develop a storable, modified freeze‐dried diet that could be used to raise host‐specific insects of conservation concern year‐round. Unlike the population trends when using natural host plant availability to feed the larvae, the population of the experimental colony did not fail during the winter after implementing the use of the freeze‐dried diet (Figure 1). The large population numbers in this period further reinforce the viability of the freeze‐dried diet to support butterfly colonies in managed care.

The Atala hairstreak larvae in our study were physically capable of eating the freeze‐dried diet, and the insects developed from larvae to chrysalis to adult. Compared to previous iterations of an artificial diet trial which utilized coontie mixed with an off‐the‐shelf insect diet, and which saw mortality rates of 50%–100% (Ardente et al., 2017), mortality was low and pupation success was high for our experimental colony. As seen in Table 1, we found no significant difference in pupation and emergence success rates for Atala butterflies raised on the modified diet versus the control diet. Insects raised on the freeze‐dried diet developed slightly more slowly than the control insects (Table 1), although we were unable to test whether this difference was significant because we did not follow the development of individual animals. We did not measure weight, length, frass, or similar aspects because Atala are gregarious animals, making separate longitudinal measurements difficult. We are not certain why the larvae fed freeze‐dried diet developed more slowly, although development time differences have been noted in other insects raised on artificial diets (e.g., Ellis, 1998; Holloway et al.; Reynolds et al., 1986; Scriber & Feeny, 1979; Timmins et al., 1988) and may be due to leaf moisture content, which was selected for practical purposes rather than by mimicking natural leaf moisture levels in fresh growth coontie. Despite the slightly slower development rate compared to the control, the freeze‐dried diet successfully sustained the Atala colony through the winter when fresh growth from their host plant was unavailable.

A steady colony population enabled consistent management practices and reintroduction efforts. The methods used to develop the freeze‐dried diet for the Atala butterfly described in this paper used to produce 3847 butterflies over an extended period of time (~2.3 years) (Figure 1). We used the freeze‐dried diet to maintain a colony of Atala butterflies year‐round and release thousands into the wild, which would not be possible otherwise due to scarcity of suitable host plant material during the winter. We therefore conclude that the freeze‐dried diet has been successful for this species and recommend its use for those interested in Atala conservation, or its application for processing other host plant material for species of conservation concern.

We suggest three major lines of follow‐up research. First, based on the success of the freeze‐dried diet with the Atala butterfly colony, the freeze‐drying method could be used to create diets for maintaining colonies of other species dependent on seasonal host plants and provide opportunities for supplementing the diet of other imperiled species in managed care. For example, rearing the endangered Miami blue butterfly requires constant fresh knicker bean, and most monarch conservation plans require milkweed planting (CEC, 2008; Caldwell et al., 2018). Specialized species like these could potentially benefit from freeze‐dried food. Second, best practices for the storage and use of this diet should be established. While we know our current stock works, we do not know its shelf life, nor have we experimented with different methods of storage besides our current practice of keeping it in a freezer. Third, data on nutritional content over time is necessary to draw stronger conclusions about the long‐term use of modified diets for butterflies. Many insects, including the Atala butterfly, acquire defensive chemicals from their host plants (Koi & Daniels, 2015). Follow‐up research could determine whether the concentrations of defensive chemicals in insects raised on artificial diet differ from insects found in the wild and populations in managed care raised on fresh host plant material. Through future research and applications, modifying diets for host‐specific butterflies can improve to better meet the needs of and conserve these sensitive species.

## 5 CONCLUSIONS

- Our methods of using modified freeze‐dried to feed larvae less than 9 mm and fresh plants to feed larger larvae were successful in maintaining a colony of Atala hairstreak butterflies
- The larvae fed on freeze‐dried diet did not differ from larvae fed fresh coontie in pupation and emergence rates
- Methods for creating this simple diet could potentially be applied to other specialized butterflies
- Potential further research for this species includes: optimal diet storage parameters, egg viability, nutrient content, toxicity of larvae, and greater detail on growth rates and weights of larvae fed on freeze‐dried diet

## CONFLICT OF INTERESTS

The authors declare that there are no conflict of interests.

## Supporting information

Supporting information.

## Data Availability

The data that support the findings of this study are available on request from the corresponding author. The data are not publicly available due to privacy or ethical restrictions. The data that support the findings of this study are available from the corresponding author upon reasonable request.
