# Supplementary material for: A modified diet to support conservation of the Atala hairstreak butterfly (Eumaeus atala Poey)
Source: Zoo Biol. 2021 Jun 14;40(5):429–35. doi: 10.1002/zoo.21628 (PMC8596422; doi:10.1002/zoo.21628)

APPENDICES AND SUPPORTING INFORMATION

**Appendix 1.** Moisture and Dry Matter content of new-growth, mature leaves, mixed-age browse, and freeze-dried coontie. Mixed-age browse is what is typically harvested to feed to the colony.

| **Coontie sample type** | **Moisture** | **Dry Matter** |
| --- | --- | --- |
| New growth leaves only | 80.9 % | 19.1 % |
| Mature leaves only | 59.8 % | 40.2 % |
| Mixed-age browse (as harvested for typical feeding) | 62.0 % | 38.0 % |
| Freeze dried leaves | 3.7 % | 96.3 % |
|  |  |  |

SUPPLEMENTARY FIGURE LEGENDS

**Supplementary Fig. 1** Freeze-dried coontie powder.

**Supplementary Fig. 2.** Materials for mixing the freeze-dried diet. Diet components (coontie powder and water) were mixed at a ratio of 1 gram powder: 4:25 grams water.

**Supplementary Fig. 3.** Larval setup. Larvae were reared on feeding trays inside of tubs. Fresh freeze-dried diet mush was added each day.

**Supplementary Fig. 4**. Modified freeze-dried fed group in experimental growth trial crossing bridges to new feeding trays. Some handling still occurred when slow larvae (example circled) failed to find food. Slow larvae were moved at the beginning of each day with brushes.

**Supplementary Fig. 5.** Flight cage set up for adults. Nectar feeders were eventually replaced with circular, flat feeders that could feed more adults. We eventually replaced the potted plant with single sprigs of leaves.

**Supplementary Fig. 6.** Control larvae fed on fresh plants for the growth trial were reared in similar tubs on clippings of young coontie leaves.

SUPPLEMENTARY FIGURES

**Supplementary Fig. 1**


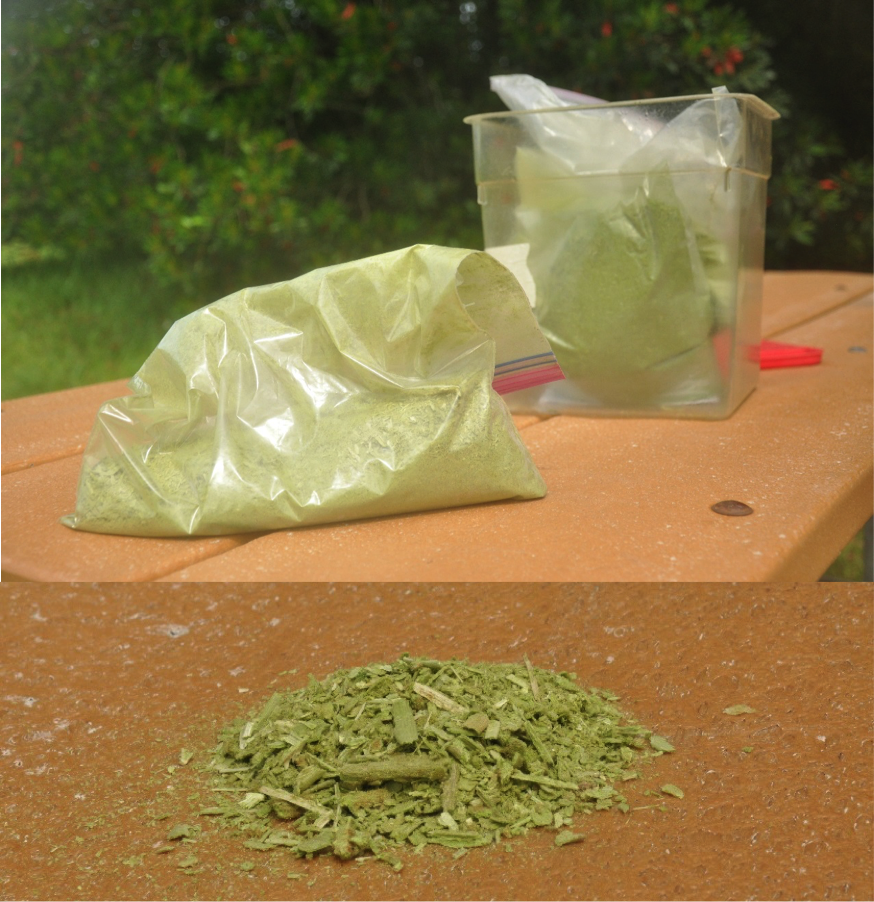


**Supplementary Fig. 2**


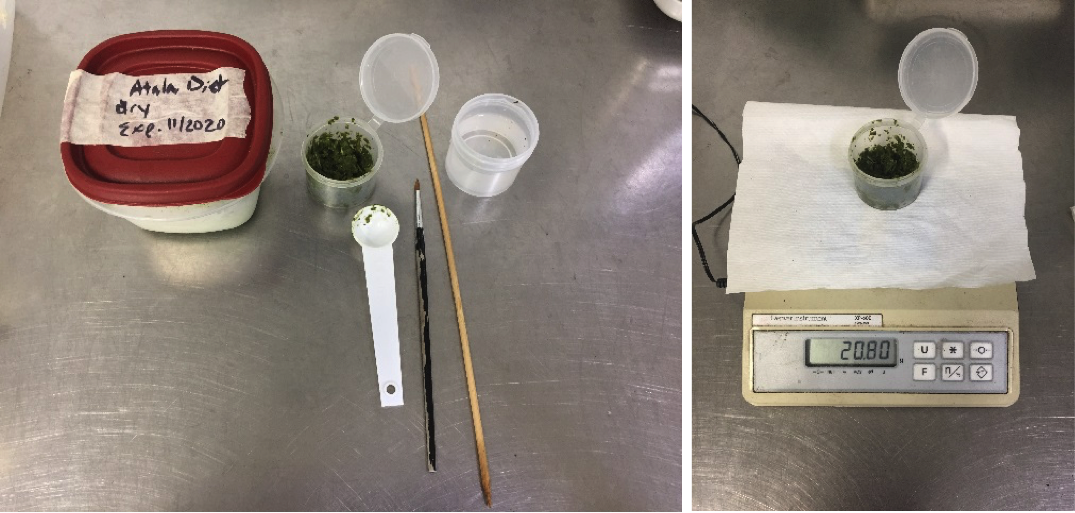


**Supplementary Fig. 3**


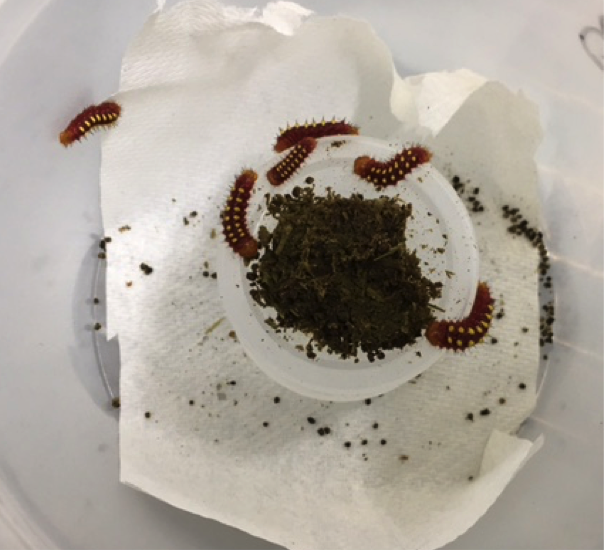


**Supplementary Fig. 4**


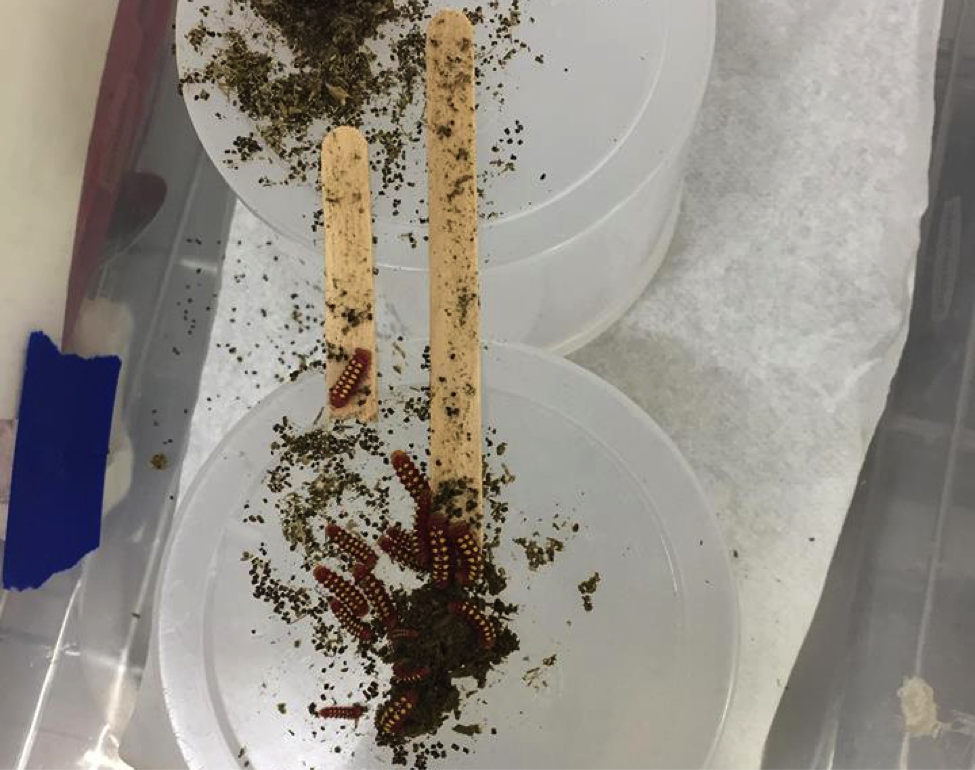


**Supplementary Fig. 5**


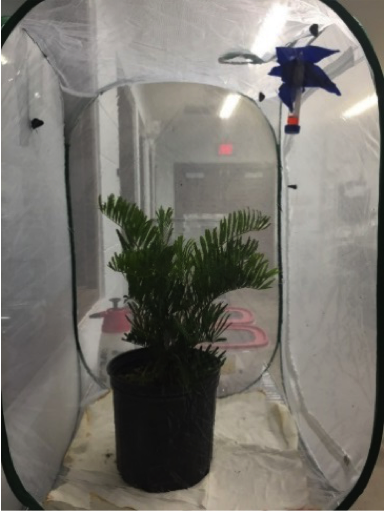


**Supplementary Fig. 6**


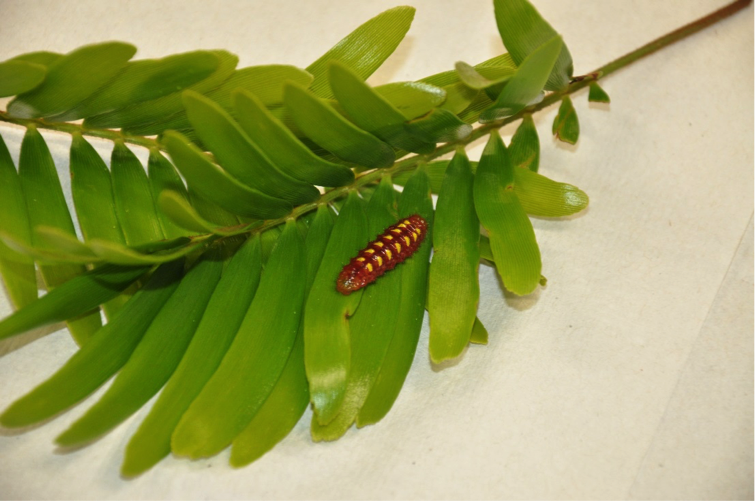

Supplement: Supplementary file 1 — Supporting information. [file ZOO-40-429-s001.docx]
